# Supplementary material for: Antibacterial activity of medicinal plants in Indonesia on Streptococcus pneumoniae
Source: PLoS One. 2022 Sep 13;17(9):e0274174. doi: 10.1371/journal.pone.0274174 (PMC9469987; doi:10.1371/journal.pone.0274174)
Supplement: S1 Fig — (DOCX) [file pone.0274174.s001.docx]

**Figure S1**. **Inhibition zones of plant extracts on *S. pneumoniae* ATCC 49619**. Extract-impregnated disc (6mg/disc) was used to obtain inhibition zone performed on sheep blood (5%) Mueller-Hinton agar. The incubation was done at 37^o^C with 5% CO_2_ for 20 hours
