# Supplementary material for: Antibacterial activity of medicinal plants in Indonesia on Streptococcus pneumoniae
Source: PLoS One. 2022 Sep 13;17(9):e0274174. doi: 10.1371/journal.pone.0274174 (PMC9469987; doi:10.1371/journal.pone.0274174)
Supplement: S3 Fig — (DOCX) [file pone.0274174.s003.docx]

**Figure S3. Ultrastructure changes on ATCC 49619 caused by *L. inermis* ethyl acetate extract**. a) untreated *Streptococcus pneumoniae.* b) ATCC 49619 treated with extract. *Streptococcus pneumoniae* was incubated with *L. inermis* extract at final concentration 2×MIC for 2 hours prior to sample block preparation. Bacterial cells were fixed with 2,5% glutaraldehyde. The observation was done at 8000 – 12000 magnification. Arrow-head; extracellular vesicle, black-triangle; cell wall disruption, white-triangle; cell membrane folding,
