# Supplementary material for: Antibacterial activity of medicinal plants in Indonesia on Streptococcus pneumoniae
Source: PLoS One. 2022 Sep 13;17(9):e0274174. doi: 10.1371/journal.pone.0274174 (PMC9469987; doi:10.1371/journal.pone.0274174)
Supplement: S5 Fig — (DOCX) [file pone.0274174.s005.docx]

**Figure S5. Ultrastructure changes on MDRSP caused by *L. inermis* ethanol extract.** a) untreated *Streptococcus pneumoniae.* b) MDRSP 2506 treated with extract. *Streptococcus pneumoniae* was incubated with *L. inermis* extract at final concentration 2×MIC for 2 hours prior to sample block preparation. Bacterial cells were fixed with 2,5% glutaraldehyde. The observation was done at 8000 – 12000 magnification. Arrow; bacterial leakage
