# Supplementary material for: Antibacterial activity of medicinal plants in Indonesia on Streptococcus pneumoniae
Source: PLoS One. 2022 Sep 13;17(9):e0274174. doi: 10.1371/journal.pone.0274174 (PMC9469987; doi:10.1371/journal.pone.0274174)
Supplement: S6 Fig — (DOCX) [file pone.0274174.s006.docx]

**Figure S6. Chromatogram of *L. inermis* ethanol extract obtained with LC MS/MS.** A 1 mg/ml plant extract diluted in sterile ddH_2_O was used for phytochemical compound detection with LC MS/MS.
