# Supplementary material for: Antibacterial activity of medicinal plants in Indonesia on Streptococcus pneumoniae
Source: PLoS One. 2022 Sep 13;17(9):e0274174. doi: 10.1371/journal.pone.0274174 (PMC9469987; doi:10.1371/journal.pone.0274174)
Supplement: Attachment — Submitted filename: Response to reviewers.docx [file pone.0274174.s012.docx]

**Rebuttal Letter**

Dear Academic Editor of the PLOSONE Journal,

We thank the editor and reviewers for their critical assessment and constructive suggestion of our manuscript. The reviewers' comments were helpful in improving the overall quality of our manuscript. We have revised our manuscript according to the comments and suggestions made by the reviewers and editors. We hope the revisions in our manuscript and the accompanying responses to the reviewers' comments will be sufficient to make our manuscript acceptable for publication in the PLOSONE Journal.

Regards,

On behalf of all authors

Wisnu Tafroji, M. Biomed

Eijkman Research Center for Molecular Biology

Jakarta – Indonesia

**Title:** Antibacterial activity of medicinal plants in Indonesia on *Streptococcus pneumoniae*

**Authors:** Wisnu Tafroji*^a,b^**,** Nur Ita Margyaningsih**^a^**, Miftahuddin Majid Khoeri^a,d^, Wisiva Tofriska Paramaiswari^a^, Yayah Winarti^a^, Korrie Salsabila^a,b^, Hanifah Fajri Maharani Putri^a^, Nurjati Chairani Siregar^a,c^ Amin Soebandrio^a,c^, Dodi Safari^a^

**Manuscript No. :** PONE-D-22-12448

**Reviewers’ comments**

**Reviewer #1**

1. There is a lack of novelty in the manuscript. Why authors did this study with plant extract? what are the photochemical compounds in these extracts? many articles published regarding this topic and what is important in this study?
   I recommend the rejection of this article.

**Authors’ response:** Thank you very much for your thought on this manuscript. We appreciate your decision on this manuscript. In general, there are many publication on describing antimicrobial activity of plant extract against bacteria, but limited to fast growing bacteria such as *Staphylococcus aureus*, *Pseudomonas aeruginosa*, *Escherichia coli* and many other bacteria. As our knowledge, publication that describes antimicrobial activity of medicinal plants on fastidious bacteria such as *Streptococcus pneumoniae* is still limited. Besides, many publications on fastidious bacteria only describe antimicrobial activity by inhibition zone. In this manuscript, we have elaborated the antimicrobial activity against *S. pneumoniae* with minimum inhibitory concentration which is more sensitive to determine antimicrobial activity as well as minimum concentration needed for further testing such as new candidate of antibiotics development. In addition, descriptions on bacterial lysis, time of killing, gene expression of toxin-antitoxin system (epsilon-zeta), and ultrastructure changes on bacterial cells appearance of *S. pneumoniae*  as well as the methods are the novelty of this manuscript since this information is not very common for recent publication.

**Reviewer #2**

1. In the manuscript entitled " Antibacterial activity of medicinal p 1 lants in Indonesia on Streptococcus pneumoniae", the authors prepared extracts from many different types of medical plants using different solvents and tested the antibacterial activity of these extracts against *Streptococcus pneumoniae*. Most conclusions are supported by appropriate results. I recommend that the manuscript can be considered to accept for publication after the following issues have been addressed:

**Authors’ response:** Thank you very much for your feedback and support on this manuscript. We have responded some issues and revised the manuscript according your suggestion for improving overall quality of this manuscript.

2. There are many grammar errors in the manuscript. Using commas as the decimal point is not an international way. For example, MIC 0,16 mg/mL is suggested to be modified as “0.16 mg/mL”. Also, the number in line 65, 400.000 is suggested to be modified as “400,000” or “400000”.

**Authors’ response:** Thank you very much for your suggestion. We have revised the manuscript according to correction and suggestion made.

3. In line 66, the authors wrote “Drug resistant Streptococcus pneumoniae was reported in Indonesia”, and in line 67, the authors wrote “many drug resistant Streptococcus pneumoniae was reported in Indonesia”. This sentence is repeated.

**Authors’ response:** Thank you very much for your feedback. We have revised the sentences to avoid repetition. We revised the addressed sentences to “Many drug resistant *Streptococcus pneumoniae* strains were reported in Indonesia such as Tetracycline resistant of *S. pneumoniae* that was reported in 2001, cotrimoxazole-resistant and tetracycline-resistant strain that were commonly reported”

4. In line 72, such as what bacteria?

Authors’ response: Thank you for your feedback. We thought that this sentence was accidentally erased during editing. We have completed the sentence as “Medicinal plants are reported having antibacterial activity against some human pathogenic bacteria such as *Staphylococcus aureus*, *Pseudomonas aeruginosa*, *Escherichia coli*, and *Enterococcus sp*”

5. In line 79, the word “were” should be deleted.

**Authors’ response:** Thank you for your suggestion. We have deleted “were” in the following line specified.

6. In the method section, line 110, the extraction was done using hexane, and followed by acetate, ethanol…, or the extraction was done in hexane, acetate, ethanol…., respectively to get different extractions?

**Authors’s response:** Thank you for your feedback. The extraction was done sequentially, in order we extracted the plant firstly with n-hexane by soaking overnight with agitation followed by filtration by using filter paper. The residue of plant filtered on filter paper was continued for extraction with ethyl acetate until filtration, this sequence was repeated for ethanol until water solvent.

7. In the method section, line 132, why did the authors choose “30 µg” vancomycin as a positive control for the disc diffusion? The authors should explain this. Otherwise, it is hard to judge if 30 µg vancomycin is a suitable control. Also, in the later tests, the authors adopted 4 µg/mL vancomycin as control. Similarly, the authors should explain why they choose “4 µg” vancomycin as a control? Why do you think it is a suitable control?

**Authors’ response:** Thank you for your suggestion. Vancomycin 30µg as well as vancomycin 4 µg/ml are chosen for positive control because the majority of *S. pneumoniae* including the MDR strain are susceptible to vancomycin specifically for a 30µg concentration according to CLSI guidelines by resulting inhibition zone ≥ 17 mm and the vancomycin 4 µg/ml that will produce clear inhibition for both ATCC and MDDR strain used in this study. I have added this additional description in the following line addressed in the comment.

8. This work used DMSO as the solvent to dissolve plant extracts and used them to do antibacterial tests. The application of these solutions might be a problem DMSO is toxic to cells. It is suggested to use safer solvents if possible.

**Authors’ response:** Thank you for your feedback. Yes, we agree with the reviewer’s comments. Therefore, we used DMSO at final concentration 1%, 10% and 100% as a solvent control for disk diffusion and microdilution to consider possible confounding factor of DMSO toxicity to bacterial cells. We found that there was no inhibition of DMSO affecting bacterial growth that we assumed there is no confounding factor affecting the results caused by DMSO toxicity.

9. In Fig. 1A, which plant extracts were used to get these data? It needs to be clarified.

**Authors’ response:** Thank you for your recommendation. The Fig. 1A is an average of inhibition zone of all plant extracts used in this study grouped according to the solvent used. It is not specified only to certain plant extracts.

10. In Fig. 3, where are the curves of 1 X MIC and 2X MIC? If these curves are overlapped with that of 10 X MIC, please describe it in the main text. If 1 X MIC and 2X are different to that of 10 X MIC, please add the data.

**Authors’ response:** Thank you very much for your feedback. Did you mean the 2x MIC curve overlapped with 10x MIC ? if yes, we have add description of overlapping curves in the caption of figure 3 instead of in the main text since we thought it will be easier for the readers. All data set supporting data presentation in this manuscript have been deposited on DOI: 10.6084/m9.figshare.20364249

11. In the Mechanism of Actions section, Bacterial lysis, the authors used the growth curves of bacteria by measuring absorbance at 595 nm, and saying bacterial lysis happened because of the decrease of the absorbance at 595 nm. Why? I do not agree with this, because it is a normal growth curves measurement. The decrease of the absorbance only shows the inhibitive effects of the drug you use, but the mechanism behind is unknown. This part needs serious revision.

**Authors’ response:** Thank you for your suggestion. We agree with reviewer’s comment on the use of 595 nm for bacterial growth measurement. The measurement is done based on the ability of 595 nm wavelength in detecting bacterial cells. Increasing of 595 nm absorbance will be concordant with increasing of bacterial cells showing bacterial growth. The inhibition of bacterial growth will result on a plateau curves instead of decreasing. Therefore, the decreasing curves showed decreasing of bacterial cell due to cell lysis since we did not observe the plateau curves before decreasing as normal growth curves had. In addition, we also confirmed bacterial lysis at 2 hours of incubation with plant extracts, when the decreasing curves were observed, under Transmission electron microscope that we found some bacterial cells lysed.

12. In Fig.5, the description of a to e should be added in the figure caption.

**Authors’ response:** Thank you for your suggestion. We have added description of a to e in the caption as you suggested.

**Reviewer #3**

1. The finding is interesting, however, there are some minor mistakes have been observed (like grammar, spellings, commas, pronunciations etc.). The authors are requested to kindly correct all those errors. Also, there is a need to update the references. over-all the manuscript looks good. Tables and graphs are fine and beautifully presented. I recommend that this manuscript can be accepted for publication after minor revisions. Congratulations to authors for this wonderful work.

**Authors’ response:** Thank you very much for your supportive comments. We have revised and rechecked the grammatical error as well as spellings, and others as you recommended.
